# Hypothesis-Testing Demands Trustworthy Data—A Simulation Approach to Inferential Statistics Advocating the Research Program Strategy

**DOI:** 10.3389/fpsyg.2018.00460

**Published:** 2018-04-24

**Authors:** Antonia Krefeld-Schwalb, Erich H. Witte, Frank Zenker

**Affiliations:** ^1^Geneva School of Economics and Management, University of Geneva, Geneva, Switzerland; ^2^Institute for Psychology, University of Hamburg, Hamburg, Germany; ^3^Department of Philosophy, Lund University, Lund, Sweden

**Keywords:** Bayes' theorem, inferential statistics, likelihood, replication, research program strategy, *t*-test, Wald criterion

## Abstract

In psychology as elsewhere, the main statistical inference strategy to establish empirical effects is null-hypothesis significance testing (NHST). The recent failure to replicate allegedly well-established NHST-results, however, implies that such results lack sufficient statistical power, and thus feature unacceptably high error-rates. Using data-simulation to estimate the error-rates of NHST-results, we advocate the *research program strategy* (RPS) as a superior methodology. RPS integrates Frequentist with Bayesian inference elements, and leads from a preliminary discovery against a (random) *H*_0_-hypothesis to a statistical *H*_1_-verification. Not only do RPS-results feature significantly lower error-rates than NHST-results, RPS also addresses key-deficits of a “pure” Frequentist and a standard Bayesian approach. In particular, RPS aggregates underpowered results safely. RPS therefore provides a tool to regain the trust the discipline had lost during the ongoing replicability-crisis.

## Introduction

Like all sciences, psychology seeks to establish *stable* empirical hypotheses, and only “methodologically well-hardened” data provide such stability (Lakatos, 1978). In analogy, data we cannot replicate are “soft.” Recent attempts to replicate allegedly well-established results of null-hypothesis significance testing (NHST), however, did broadly fail. As did the five preregistered replications, conducted between 2014 and 2016, reported in *Perspectives of Psychological Science* (Alogna et al., 2014; Cheung et al., 2016; Eerland et al., 2016; Hagger et al., 2016; Wagenmakers et al., 2016). This implies that the error-proportions of NHST-results generally are too large. For many more replication attempts should otherwise have succeeded.

We can partially explain the replication failure of NHST-results by citing questionable research practices that inflate the Type-I error probability (false positives), as signaled by a large α-error (Nelson et al., 2018). If researchers collect undersized samples, moreover, then this raises the Type-II error probability (false negatives), as signaled by a large β-error. (The latter implies a lack of sufficient test-power i.e., 1 – β-error). *Ceteris paribus*, as these errors increase, the replication-probability of a true hypothesis decreases, thus lowering the chance that a replication attempt obtains a similar data-pattern as the original study. Since NHST remains *the* statistical inference strategy in empirical psychology, many today (rightly) view the field as undergoing a replicability-crisis (Erdfelder and Ulrich, 2018).

It adds severity that this crisis extends beyond psychology—to *medicine* and *health care* (Ioannidis, 2014, 2016), *genetics* (Alfaro and Holder, 2006), *sociology* (Freese and Peterson, 2017), and *political science* (Clinton, 2012), among other fields (Fanelli, 2009)—and affects each field as a whole. A 50% replication-rate in *cognitive* psychology vs. a 25% replication-rate in *social* psychology (Open Science Collaboration, 2015), for instance, merely makes the first subarea appear more crisis-struck. Since all this keeps from having too much *trust* in our published empirical results, the term “confidence-crisis” is rather apt (Baker, 2015; Etz and Vandekerckhove, 2016).

The details of how researchers standardly employ NHST coming under doubt has sparked renewed interest in statistical inference. Indeed, many researchers today self-identify as either Frequentists or Bayesians, and align with a “school” (Fisher, Neyman-Pearson, Jeffreys, or Wald). However, statistical inference as a whole offers no more (nor less) than a probabilistic logic to estimate the support that a hypothesis, *H*, receives from data, *D* (Fisher, 1956; Hacking, 1965; Edwards, 1972; Stigler, 1986). This estimate is technically an inverse probability, known as the *likelihood, L*(*H*|*D*), and (rightly) remains central to Bayesians.

An important precondition for calculating *L*(*H*|*D*) is the *probability* of *D* given *H, p*(*D*,*H*). Unlike *L*(*H*|*D*), we cannot determine *p*(*D*,*H*) other than by induction over data. This (rightly) makes *p*(*D*,*H*) central to Frequentists. Testing *H* against *D*—in the sense of estimating *L*(*H*|*D*)—thus presupposes induction, but nevertheless remains distinct conceptually. Indeed, the term “test” in “NHST” misleads. For NHST tests only *p*(*D*,*H*), but not *L*(*H*|*D*). This may explain why publications regularly over-report an NHST-result as *supporting* a hypothesis. Indeed, many researchers appear to misinterpret NHST as the statistical hypothesis-testing method it emphatically is not.

To clarify why testing *p*(*D*,*H*) conceptually differs from testing *L*(*H*|*D*), this article compares NHST with the *research program strategy* (RPS), a hybrid-approach that integrates Frequentist with Bayesian statistical inference elements (Witte and Zenker, 2016a,b, 2017a,b). As “stand-ins” for real empirical data, we here simulate the distribution of a (dependent) variable in hypothetical treatment- and control-groups to simulate that variable's arithmetic mean in both groups. Our simulated data are sufficiently similar to data that actual studies would collect for purposes of assessing whether an independent, categorical variable (e.g., an experimental manipulation) significantly influences a dependent variable. Therefore, simulating the parameter-range over which hypothetical data are sufficiently replicable does estimate whether actual data are stable, and hence *trustworthy*.

We outline RPS [section The Research Program Strategy (RPS)], detail three statistical measures (section Three Measures), explain purpose, method, and the key-result of our simulations (section Simulations), offer a critical discussion (section Discussion), then compare RPS to a “pure” Frequentist and a standard Bayesian approach (section Frequentism Vs. Bayesianism Vs. RPS), and finally conclude (section Conclusion). As supplementary material, we include the R-code, a technical appendix, and an online-app to verify quickly that a dataset is sufficiently stable^1^.

## The research program strategy (RPS)

With the construction of empirical theories as its main aim, RPS distinguishes the *discovery context* from the *justification context* (Reichenbach, 1938). The discovery context readily lets us induce a data-subsuming hypothesis *without* requiring reference to a theoretical construct. Rather, discerning a non-random data-pattern, as per *p*(*D*,*H*_0_) < α ≤ 0.05, here sufficiently warrants accepting the *H*_1_-hypothesis that is a best fit to *D* as a *data-abbreviation*. Focusing on non-random effects alone, then, discovery context research is fully data-driven.

In the justification context, by contrast, data shall firmly test a theoretical *H*_1_-hypothesis, i.e., verify or falsify the *H*_1_ probabilistically. A hypothesis-test must therefore *pitch* a theoretical *H*_1_-hypothesis either against the (random) *H*_0_-hypothesis, or against some substantial hypothesis besides the *H*_1_ (i.e., *H*_2_, …, *H*_*n*__−1_, *H*_*n*_). Were the *H*_1_-hypothesis we are testing *indistinct* from the data-abbreviating *H*_1_, however, then data once employed to induce the *H*_1_ now would confirm it, too. As this would *level* the distinction between theoretical and inductive hypotheses, it made “hypothesis-testing” an empty term. Hence, justification context research *must* postulate a theoretical *H*_1_.

Having described and applied RPS elsewhere (Witte and Zenker, 2016a,b, 2017a,b), we here merely list the six (individually necessary and jointly sufficient) RPS-steps to a probabilistic hypothesis-verification^2^.

**Preliminary discovery**
  The first step discriminates a random fluctuation (*H*_0_) from a systematic empirical observation (*H*_1_), measured either by the *p*-value (Fisher) or the α-error (Neyman-Pearson). Under accepted errors, we achieve a *preliminary H*_1_*-discovery* if the empirical effect sufficiently deviates from a random event.
**Substantial discovery**
  Neyman-Pearson test-theory (NPTT) states the probability that a preliminary discovery is replicable as the (1–β-error), aka test-power. If we replicate a preliminary discovery while α- and β-error (hereafter: α, β) remain sufficiently small, a preliminary *H*_1_-discovery turns into a *substantial H*_1_*-discovery*.
**Preliminary falsification**
  A substantial *H*_1_-discovery may entail that we thereby *preliminarily falsify* the *H*_0_ (or another point-hypothesis). As the falsification criterion, we propose that the likelihood-ratio of the theoretical effect-size *d* > 0, postulated by the *H*_1_, and of a null-effect *d* = 0, postulated by the *H*_0_, i.e., $\frac{L\left(d>0|D\right)}{L\left(d=0|D\right)}$, must exceed Wald's criterion $\frac{\left(1-\beta \right)}{\alpha }$ (Wald, 1943).
**Substantial falsification**
  A preliminary *H*_0_-falsification turns into a *substantial H*_0_*-falsification* if the likelihood-ratio of all theoretical effect-sizes that exceed the minimum theoretical effect-size *d* > δ = *dH*_1_ – *dH*_0_, and of the *H*_0(*d*=0)_, i.e., $\frac{L\left(d>\delta |D\right)}{L\left(d=0|D\right)}$, exceeds the same criterion, i.e., $\frac{\left(1-\beta \right)}{\alpha }$.
**Preliminary verification**
  We achieve a *preliminary H*_1_*-verification* if the likelihood ratio of the point-valued *H*_1(*d* = δ)_ and the *H*_0(*d*=0)_ exceeds, again, $\frac{\left(1-\beta \right)}{\alpha }$.
**Substantial verification**
  Having preliminarily verified the *H*_1(*d*=δ)_ against the *H*_0(*d*=0)_, we now test how similar δ is to the empirical (“observed”) effect-size's maximum-likelihood-estimate, MLE_(*d*emp)_. As our verification criterion, we propose the ratio of both likelihood-values (i.e., the maximal ordinate of the normal distribution divided by its ordinate at the 95% interval-point), which is approximately 4 (see next section). If δ's likelihood falls within the 95%-interval centered on MLE_(*d*emp)_, then we achieve a *substantial H*_1_*-verification*. This means we now accept “*H*_1(*d*=δ)_” as shorthand for the effect-size our data corroborate statistically.

**Figure 1:**
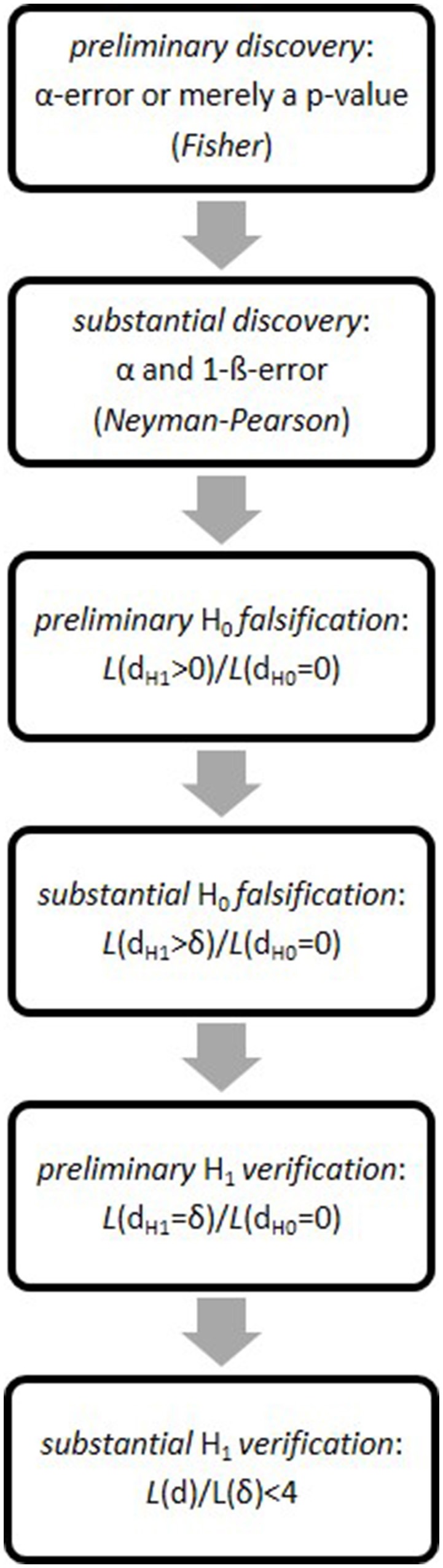
The six steps of the research program strategy (RPS).

RPS thus starts in the discovery context by using *p*-values (Fisher), proceeds to an optimal^3^ test of a non-zero effect-size against either a random-model or an alternative model (Neyman-Pearson), and reaches—entering into the justification context—a statistical verification of a theoretically specified effect-size based on probably replicable data^4^ (see Figure 1). All along, of course, we must assume accepted α**- and β**-error.

In what we call the *data space*, RPS-steps 1 and 2 thus evaluate probabilities; RPS-steps 3–5 evaluate likelihoods in the *hypotheses space*; and RPS-step 6 returns to the data space. For data alone determine if the point-hypothesis from RPS-step 5 is substantially verified at RPS-step 6, or not. As if in a circle, then, RPS balances threes steps in the data space (1, 2, 6) with three steps in the hypotheses space (3, 4, 5).

Importantly, individual research groups rarely command sufficient resources to collect a sufficiently large sample that achieves the desirably low error-rates a well-powered study requires (see note 3). To complete all RPS-steps, therefore, groups must coordinate *joint* efforts, which requires a method to aggregate underpowered studies safely (We return to this toward the end of our next section).

Since RPS integrates Frequentist with Bayesian statistical inference-elements, the untrained eye might discern an arbitrary “hodgepodge” of methods. Of course, the Frequentist and Bayesian schools both harbor advantages and disadvantages (Witte, 1994; Witte and Kaufman, 1997; Witte and Zenker, 2017b). For instance, Bayesian statistics allows us to infer hypotheses from data, but normally demands greater effort than using Frequentist methods. The simplicity and ubiquity of Frequentist methods, by contrast, facilitates the application and communication of research results. But it also risks to neglect assumptions that affect the research process, or to falsely interpret such statistical magnitudes as confidence intervals or *p*-values (Nelson et al., 2018). Decisively, however, narrowly sticking to any one school would simply avoid attempting to integrate each school's best statistical inference-elements into an all-things-considered best strategy. RPS does just this.

RPS motivates the selection of these elements by its main goal: to construct informative empirical theories featuring precise parameters and hypotheses. As RPS-step 1 exhausts the utility of α, or the *p*-value (*preliminary discovery*), for instance, β additionally serves at RPS-step 2 (*substantial discovery*). In general, RPS deploys inference elements at any subsequent step (e.g., the effect size at RPS-step 2–5; confidence intervals at RPS-step 6) to sequentially increase the information of a preceding step's focal result.

Unlike what RPS may suggest, of course, the actual research process is not linear. Researchers instead stipulate both the hypothesis-content and the theoretical effect-size freely. Nevertheless, a hypothesis-test deserving its name—one estimating *L*(*H*|*D*), that is—requires replicable rather than “soft” data, for such data alone can meaningfully induce a *stable* effect-size.

RPS therefore measures three qualities: induction quality of data, as well as falsification quality and verification quality of hypotheses, to which we now turn.

## Three measures

This section defines three measures and their critical values in RPS. The first measure estimates how well data sustain an induced parameter; the second and third measure estimate how well replicable data undermine and, respectively, support a hypothesis^5^.

*Def. induction quality*: Based on NPTT, we measure induction quality as α and β, given a fixed sample size, *N*, and two point-valued hypotheses, *H*_0_ and *H*_1_, yielding the effect-size difference *dH*_1_ – *dH*_0_ = δ.

The measure presupposes the effect-size difference *dH*_1_ – *dH*_0_ = δ, for otherwise we could not determine test-power (1–β).

Since induction quality pertains to the (experimental) conditions under which one collects data, the measure qualifies an empirical setting's *sensitivity*. Whether a setting is acceptable, or not, rests on convention, of course. RPS generally promotes α = β = 0.05, or even α = β = 0.01, as the right sensitivity (see section Frequentism Vs. Bayesianism Vs. RPS). By contrast, α = 0.05 and β = 0.20 are normal today. Since $\frac{\beta }{\alpha }=\frac{0.20}{0.05}=4$, this makes it four times more important to discover an effect than to replicate it—an imbalance that counts toward explaining the replicability-crisis.

A decisive reason to instead equate both errors (α = β) is that this avoids a bias *pro* detection (α) and *contra* replicability (1–β). Given acceptable induction quality, a *substantial discovery* thus arises if the probability of data passes the critical value $\frac{\left(1-\beta \right)}{\alpha }$. Under α = β = 0.05, for instance, we find that $\frac{\left(1-\beta \right)}{\alpha }=\frac{0.95}{0.05}=19$. Hence, for the *H*_1_ to be statistically significantly more probable than the *H*_0_, we have it that *p*(*H*_1_, *D*) = 19 × *p*(*H*_0_, *D*).

Thus, we evidently can fully determine induction quality *prior* to data-collection for hypothetical data. Therefore, the measure says nothing about the focal outcome of a hypothesis-test. As we evaluate *L*(*H|D*) in the justification context, by contrast, the same measure nevertheless quantifies the trust that actual data deserve or—as the case may be—require.

*Def. falsification quality*: Based on Wald's theory, we measure falsification quality as the likelihood-ratio of all hypotheses the effect-size of which exceeds either the *H*_0_ (preliminary falsification) or δ (substantial falsification), and the point-valued *H*_0_, i.e., *L*(*d* > 0|*D*)/*L*(*d* = 0|*D*). Our proposed falsification-threshold (1−β)/α thus depends on induction quality of data.

The falsification quality measure rests on both the *H*_1_ and a fixed amount of actual data. It comparatively tests the point-valued *H*_0_ against all point-alternative hypotheses that exceed *d H*_1_–*d H*_0_ = δ. For instance, α = β = 0.05 obviously yields the threshold 19 (or log 19 = 2.94); α = β = 0.01 yields 99 (log 99 = 4.59), etc^6^. Since it is normally unrealistic to set α = β = 0, “falsification” here demands a statistical sense, rather than one grounding in an observation a deterministic law cannot subsume. Thus, a statistical falsification is fallible rather than final.

The same holds for verification:

*Def. verification quality*: Again based on Wald's theory, we measure verification quality as the likelihood-ratio of a point-valued *H*_1_ and a substantially falsified *H*_0_. The threshold for a *preliminary verification* is again $\frac{\left(1-\beta \right)}{\alpha }$ (thus, too, depends on induction quality of data). As the threshold for a *substantial verification*, we propose the value 4.

To explain this value, RPS views a *H*_1_-verification as preliminary if the maximum-likelihood-estimate (MLE) of data falls below the ratio of the maximum corroboration, itself determined via a normal curve's maximal ordinate, viz., 0.3989, and the ordinate at the 95%-interval centered on the maximum, viz., 0.10. As our confirmation threshold, this yields ≈4. Hence, a ratio <4 sees the theoretical parameter lie inside the 95%-interval. RPS would thus achieve a *substantial verification*.

Following Popper (1959), many take hypothesis-verification to be impossible in a deterministic sense. Understood probabilistically, by contrast, even a substantial verification of one point-valued hypothesis against another such hypothesis is error-prone (Zenker, 2017). The non-zero proportion of false negative decisions thus keeps us from verifying even the best-supported hypothesis absolutely. We can therefore achieve at most *relative* verification.

Assume we have managed to verify a parameter preliminarily. If the MLE now deviates sufficiently from that parameter's original theoretical value, then we must either modify the parameter accordingly, or may otherwise (deservedly) be admonished for ignoring experience. The MLE thus acts as a stopping-rule, signaling when we may (temporarily) accept a theoretical parameter as substantially verified.

The six RPS steps thus obtain a parameter we can trust *to the extent* that we accept the error probabilities. Unless strong reasons motivate doubt that our data are faithful, indeed, the certainty we invest into this parameter *ought* to mirror (1–β), i.e., the replication-probability of data closely matching a true hypothesis (Miller and Ulrich, 2016; Erdfelder and Ulrich, 2018).

Before sufficient amounts of probably replicable data arise in praxis, however, we must normally *integrate* various studies that each fail the above thresholds. RPS's way of integration is to add the log-likelihood-ratios of two point-hypotheses, each of which is “loaded” with the same prior probability, *p*(*H*_1_) = *p*(*H*_0_) = 0.50. Also known as log-likelihood-addition, RPS thus aggregates data of *insufficient* induction quality by relying on the well-known equation:

$$\frac{L(H_{1}|D)}{L(H_{0}|D)}=\frac{p(H_{1})p(D,H1)}{p(H_{0})p(D,H_{0})}$$

We proceed to simulate select values from the full parameter-range of possible RPS-results. These values are diverse enough to extrapolate to implicit values safely. The subsequent sections offer a discussion and then compare RPS to alternative methodologies.

## Simulations

### Overview

Using R-software, we simulate data for hypothetical treatment- and control-groups, calculate the group-means, and then compare these means with a *t*-test. While varying both induction quality of data and the effect-size, we simulate the resulting error rates. Since the simulated error-*proportions* of a *t*-test approximate the error-*probability* of data, this determines the parameter-range over which empirical results (such as those that RPS's six steps obtain) are *stable*, and hence trustworthy.

In particular, we estimate:

- the necessary sample size, *N*_MIN_, in order to register, under (1–β), the effect-size δ as a statistically significant deviation from random^7^;
- the *p*-value, as the most commonly used indicator in NHST;
- the likelihood that the empirical effect-size *d*_(emp)_ exceeds the postulated effect-size δ, i.e., *L*(*d* > δ|*D*), as a measure of substantial falsification;
- the likelihood of the *H*_0_, i.e., *L*(δ = 0|*D*), as a measure of type I and type II errors;
- the likelihood of the *H*_1_, i.e., the true effect-size *L*(δ|*D*), as a measure of preliminary verification;
- the maximum-likelihood-estimate of data, MLE(*x*), when compared to the likelihood of the *H*_1_, as a measure of substantial verification.

We conduct five simulations. Simulations 1 and 2 estimate the probability of true positive and false negative results as a function of the effect-size and test-power. Our significance level is set to α = 0.05, respectively to α = 0.01. Simulation 3 estimates the probability of false positive results. The remaining two simulations address engaging with data in *post-hoc* fashion. Simulation 4 evaluates shedding 10% of data that *least* support the focal hypothesis. To address research groups' *individual* inability to collect the large samples that RPS demands, Simulation 5 mimics collaborative research by adding the log-likelihood-ratios of underpowered studies.

### Simulation 1

#### Purpose

Simulation 1 manipulates the test-power and the true effect-size to estimate the false negative error-rates (respectively the true positive rate) throughout RPS's six steps.

#### Method

We manipulate 16 datasets that each contain 100 samples of identical size and variance. We represent a sample by the mean of a normally distributed variable in two independent groups (treatment and control), summarized with the test-statistic *t*. Between these 16 datasets, we vary the effect-size δ = [0.01, 0.2, 0.5, 0.8], and thus vary the difference between the group-means. We also vary test-power (1–β) = [0.4, 0.5, 0.8, 0.95], and thus let induction quality range from “very poor,” i.e., (1–β) = 0.4, to “medium,” i.e., (1–β) = 0.95. Under α = 0.05 (one-sided), we estimate *N*_MIN_ to meet the respective test-power (Simulation 2 tightens the significance level to α = 0.01).

#### Results and discussion

For both the experimental and the control group, Table 1 lists *N*_MIN_ to register the effect-size δ as a statistically significant deviation from random (*substantial discovery*). Generally, given constant test-power (1–β), the smaller (respectively larger) δ is, the larger (smaller) is *N*_MIN_. This shows how *N*_MIN_ depends on β.

**Table 1:** The estimated minimum sample size for a two sample *t*-test as a function of test-power (1–β) and effect size δ, given α = 0.05.

	***δ***			
**(1–β)**	**0.01**	**0.2**	**0.5**	**0.8**
0.4	38,726	97	15	6
0.5	54,111	135	22	8
0.8	123,651	309	49	19
0.95	216,443	541	87	34

For the sample sizes in Table 1, moreover, Table 2 states the proportion of *p*-values that fall below α = 0.05, given a test-power value. This estimates the probability of a *substantial discovery*. As the standard deviation of the *p*-value here indicates, we retain a large variance across samples especially for data of low induction quality.

**Table 2:** The proportion *P* of substantial discoveries, indicated by *p*-values below the significance level α = 0.05, as a function of the effect-size δ and test-power (1–β).

	***δ***							
	**0.01**		**0.2**		**0.5**		**0.8**	
**(1–β)**	***P*(*p* ≤ α)**	**σ(*p*)**	***P*(*p* ≤ α)**	**σ(*p*)**	***P*(*p* ≤ α)**	**σ(*p*)**	***P*(*p* ≤ α)**	**σ(*p*)**
0.4	0.40	0.23	0.41	0.17	0.31	0.20	0.30	0.19
0.5	0.56	0.14	0.42	0.17	0.49	0.16	0.39	0.17
0.8	0.84	0.07	0.85	0.09	0.76	0.10	0.78	0.10
0.95	0.95	0.03	0.98	0.04	0.98	0.02	0.95	0.03

*P(p < α) = proportion of significant results; σ(p) = standard deviation of p-value*.

**Table 3:** The proportion of substantial falsifications and preliminary verifications, as indicated by the respective likelihood ratio (*LR*) meeting or exceeding the threshold $LR\geq \frac{\left(1-\beta \right)}{\alpha }$.

**$P\left(LR\geq \frac{\left(1-\beta \right)}{\alpha }\right)$**	**Substantial falsification**				**Preliminary verification**			
	$\frac{L\left(d>\delta |D\right)}{L\left(d=0|D\right)}$				$\frac{L\left(d=\delta |D\right)}{L\left(d=0|D\right)}$			
	***δ***							
**(1*−β*)**	**0.01**	**0.2**	**0.5**	**0.8**	**0.01**	**0.2**	**0.5**	**0.8**
0.4	0.12	0.15	0.11	0.10	0.25	0.17	0.22	0.05
0.5	0.26	0.15	0.19	0.14	0.43	0.23	0.20	0.11
0.8	0.67	0.60	0.60	0.54	0.50	0.57	0.59	0.52
0.95	0.95	0.89	0.83	0.83	0.74	0.76	0.81	0.78

*LR, likelihood ratio; D, data*.

As with Table 1, Table 2 shows that the larger the test-power value is, the larger is the proportion of *substantial* discoveries, *ceteris paribus*. We obtain a similar result when estimating the probability of a *substantial falsification* or a *preliminary verification*, as per the likelihood-ratios $\frac{L\left(d>0|D\right)}{L\left(d=0|D\right)}$ and $\frac{L\left(d=\delta |D\right)}{L\left(d=0|D\right)}$ meeting the threshold $\frac{\left(1-\beta \right)}{\alpha }$.

In case of a *preliminary verification*, however, we obtain a larger proportion of false negative results than in case of a substantial falsification. For in verification we narrowly test a point-valued *H*_0_ against a *point*-valued *H*_1_. Whereas in falsification we test a point-valued *H*_0_ against an *interval H*_1_. Therefore, the verification criterion is “less forgiving” than the falsification criterion.

**Figure 2:**
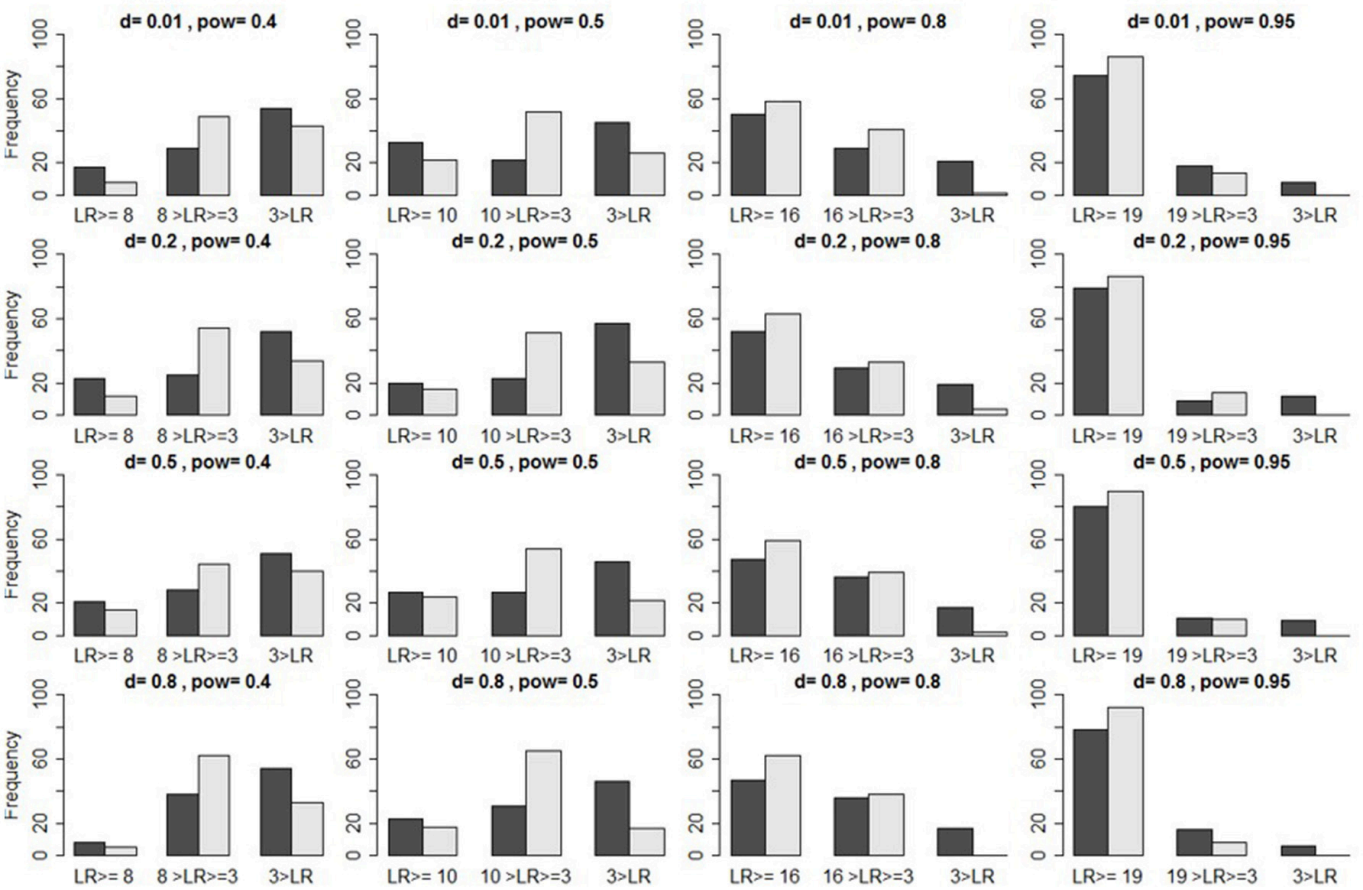
Illustration of true positives. Bar plots indicate the frequencies of likelihood ratios ($\frac{L\left(d>\delta |D\right)}{L\left(d=0|D\right)}$ set in light gray, and $\frac{L\left(d=\delta |D\right)}{L\left(d=0|D\right)}$ in dark gray) that, respectively, fall *above* the criterion $\frac{\left(1-\beta \right)}{\alpha }$ (two leftmost bars), *between* this criterion and three (two middle bars), and *below* three (two rightmost bars), as a function of induction quality of data, provided the *H*_1_ is true, under α = 0.05 [itself defined via *d* and (1–β), the latter here abbreviated as “pow”].

Using bar plots to illustrate the distribution of likelihood-ratios (*LR*s) for a preliminary verification, Figure 2 shows that *LR*s often fall below the threshold $\frac{\left(1-\beta \right)}{\alpha }$. However, if data are only of medium induction quality (α = β = 0.05), we find a large proportion of *LR*s > 3. We should therefore not immediately reject the *H*_1_, if $\frac{\left(1-\beta \right)}{\alpha }$ < *LR* > 3, because *LR* > 3 indicates some evidence for *H*_1_. Instead, we should supply additional data before evaluating the *LR*. If we increase the sample by 50% of its original size, *N*/2, for instance, but the *LR* still falls below the threshold, then we may add yet another *N*/2, and only then sum the log-*LR*s. If this too fails to yield a preliminary *H*_1_-verification (or a *H*_0_-verification), then we may still use this empirical result as a parameter-estimate which future studies might test.

An important caveat is that the likelihood-ratio measures the distance between data and hypothesis only *indirectly*. Even though the likelihood steadily increases as the mean of data approaches the effect-size that the *H*_1_ postulates, we cannot infer this distance from the *LR alone*, but must study the distribution itself. For otherwise, even if $LR\geq \frac{\left(1-\beta \right)}{\alpha }$, we would risk verifying the *H*_1_ although the observed mean of data does *not* originate with the *H*_1_-distribution, but with a distinct distribution featuring a different mean.

Moving beyond RPS-step 5, we can only address this caveat adequately by constraining the data-points that *substantially verify* the *H*_1_ to those lying in an acceptable area of variance around the *H*_1_. Table 4 reports the proportion of preliminarily *H*_1_-verifying samples that now *fail* the criterion for a *substantial H*_1_*-verification*, and thus amount to additional false negatives. We can reduce these errors by increasing the sample size, which generally reduces the error-probabilities.

**Table 4:** The proportion of preliminary verifications as per $LR\geq \frac{\left(1-\beta \right)}{\alpha }$, given the empirical effect-size *d* lies outside the interval comprising 95% of expected values placed around the *H*_1_, where $\frac{L\left(d|D\right)}{L\left(d=\delta |D\right)}>\frac{pdf\left(P_{50}|d\right)}{pdf\left(P_{95}|d\right)}>4$.

**$P\left(LR\geq \frac{\left(1-\beta \right)}{\alpha }\cap \frac{L\left(d|D\right)}{L\left(d=\delta |D\right)}>4\right)$**	***$\frac{L\left(d=\delta |D\right)}{L\left(d=0|D\right)}$***			
	***δ***			
**(1–β)**	**0.01**	**0.2**	**0.5**	**0.8**
0.4	0.07	0.03	0.02	0.00
0.5	0.08	0.05	0.04	0.00
0.8	0.05	0.04	0.05	0.04
0.95	0.05	0.02	0.04	0.02

*pdf, Probability density function; P_50_/P_95_, 50th/95th percentile*.

To account for the decrease in β after constraining the sample size in PRS-step 5, of course, the value of the threshold $\frac{\left(1-\beta \right)}{\alpha }$ now is higher, too. Hence, meeting it becomes more demanding. RPS-step 6 nevertheless increases our certainty that the data-mean originates with the hypothesized *H*_1_-distribution, and so increases our certainty in the theoretical parameter.

Table 5 states the proportion of datasets that successfully complete RPS's six steps, i.e., preliminary and substantial discovery (steps 1, 2) as well as preliminary and substantial falsification and verification (steps 3–6). For data of low to medium induction quality, we retain a rather large proportion of false negatives.

**Table 5:** The proportion of substantial verifications, after substantial discoveries and subsequent preliminary verifications were obtained, given the *H*_0_ had been substantially falsified.

	***δ***			
**(1–β)**	**0.01**	**0.2**	**0.5**	**0.8**
0.4	0.09	0.05	0.04	0.03
0.5	0.20	0.14	0.20	0.15
0.8	0.53	0.47	0.53	0.56
0.95	0.67	0.75	0.73	0.79

### Simulation 2

#### Purpose

To reduce the proportion of false negatives, as we saw, we must increase induction quality of data. Simulation 2 illustrates this by lowering the error-rates.

#### Method

Repeating the procedure of Simulation 1, but having tightened the error-rates from α = β = 0.05 to α = β = 0.01, we consequently obtain test-power (1–β) = 0.99. This also tightens the threshold from *LR* > 19 to *LR* > 99. We drop the smallest effect-size of Simulation 1 (δ = 0.01), for (1–β) = 0.99, after all, makes *N*_MIN_ = 432,952 unrealistically large (see note 3). Simulation 2 therefore comprises three datasets (each with 100 samples) and manipulates the effect-size as δ = [0.2, 0.5, 0.8].

#### Results and discussion

For these three effect sizes, Table 6 states *N*_MIN_ under α = β = 0.01. Again, the larger (smaller) the effect is, the smaller (larger) is *N*_MIN_. Simulated *p*-values continue to reflect the test-power value almost perfectly (see Table 7). Further, the proportion of preliminary verifications and substantial falsifications (see Table 8) approaches the proportion of substantial discoveries (see Table 7).

**Table 6:** Sample size for a *t*-test as a function of δ, given α = β = 0.01.

	***δ***		
	**0.2**	**0.5**	**0.8**
*N*	1,082	173	68

**Table 7:** The proportion of substantial discoveries (indicated by the *p*-value) as a function of δ, given α = β = 0.01.

***δ***					
**0.2**		**0.5**		**0.8**	
**_*P*(*p* ≤ α)_**	**σ(*p*)**	**_*P*(*p* ≤ α)_**	**σ(*p*)**	**_*P*(*p* ≤ α)_**	**σ(*p*)**
1	<0.001	0.98	0.004	0.99	0.002

**Table 8:** The proportion of substantial falsifications and preliminary verifications, indicated by the respective *LR*, as a function of δ under α = β = 0.01.

	**Substantial falsification**			**Preliminary verification**		
**$P\left(LR\geq \frac{\left(1-\beta \right)}{\alpha }\right)$**	***$\frac{L\left(d>\delta |D\right)}{L\left(d=0|D\right)}$***			***$\frac{L\left(d=\delta |D\right)}{L\left(d=0|D\right)}$***		
	***δ***					
	0.2	0.5	0.8	0.2	0.5	0.8
	0.97	0.97	0.98	0.90	0.95	0.92

Under high induction quality of data, also the proportion of false negative verifications now is acceptable. When applying the corroboration criterion for a substantial verification, we thus retain only a very small number of *additional* false negative verifications (see Table 9).

**Table 9:** The proportion of preliminary verifications as per $LR\geq \frac{\left(1-\beta \right)}{\alpha }$, where the empirical effect size *d*, however, lies outside the area spanned by the 95%-interval of expected values centered on the *H*_1_, and where $\frac{L\left(d|D\right)}{L\left(d=\delta |D\right)}>\frac{pdf\left(P_{50}|d\right)}{pdf\left(P_{95}|d\right)}>4$.

**$P\left(LR\geq \frac{\left(1-\beta \right)}{\alpha }\cap \frac{L\left(d|D\right)}{L\left(d=\delta |D\right)}>4\right)$**	***$\frac{L\left(d=\delta |D\right)}{L\left(d=0|D\right)}$***		
	***D***		
	0.2	0.5	0.8
	0.06	0.04	0.04

*pdf, Probability density function; P_50_/P_95_ = 50th/95th percentile*.

Table 10 reports the proportion of simulated datasets that successfully complete RPS-steps 3–6 in the justification context (preliminary *H*_0_-falsification to substantial *H*_1_-verification). As before, increasing induction quality of data decreases the proportion of false negative results.

**Table 10:** The proportion of substantial verifications (subsequent to achieving substantial discoveries and preliminary verifications), given that the *H*_0_ was substantially falsified under α = β = 0.01.

	***δ***		
**(1–β)**	**0.2**	**0.5**	**0.8**
0.99	0.86	0.86	0.91

### Simulation 3

#### Purpose

We have so far estimated the probability of true positive and false negative results as per the *LR* and the *p*-value. To estimate also the probability of false positive results, Simulation 3 assumes hypothetical effect-sizes and sufficiently large samples to accord with simulated test-power values.

#### Method

Simulating four datasets (100 samples each), Simulation 3 matches the sample-size to the test-power values (1–β) = [0.4, 0.5, 0.8, 0.95] for a *hypothetical* effect-size δ = 0.2. In all datasets, the simulated *true* effect-size is δ = 0.

#### Results and discussion

**Figure 3:**
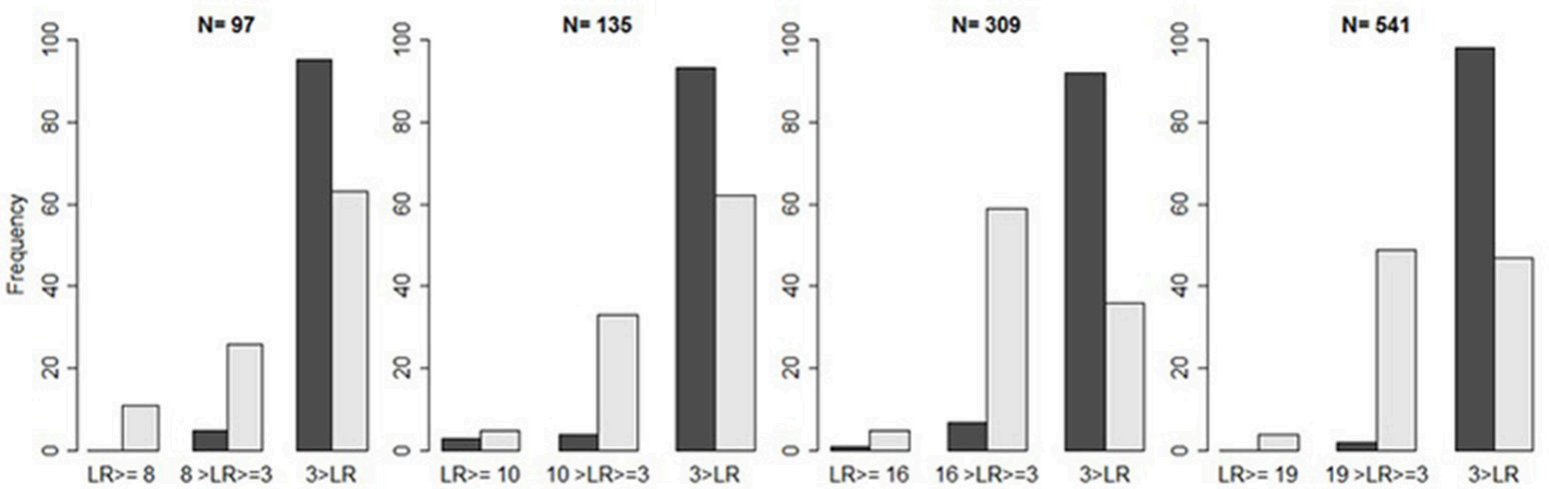
Illustration of false positives. Bar plots indicate the frequency of likelihood ratios ($\frac{L\left(d>\delta |D\right)}{L\left(d=0|D\right)}$ in light gray and $\frac{L\left(d=\delta |D\right)}{L\left(d=0|D\right)}$ in dark gray) repeatedly falling *above* the criterion $\frac{\left(1-\beta \right)}{\alpha }$, *between* the criterion and three, and *below* three, as a function of the sample size, provided the *H*_0_ is true.

Table 11 shows that simulated *p*-values reflect our predefined significance level α = 0.05. At this level, a *substantial falsification* leads to a similar proportion of false positive results as a *substantial discovery*. By contrast, a *preliminary verification* decreases the proportion of false positive results to almost zero (see Table 12). Applying the *substantial verification*-criterion even further decreases the probability of false positive results (see Figure 3).

**Table 11:** The proportion of false positives, where the sample size, *N*, is obtained by a priori power analysis, given δ = 0.2 and where (1–β) = [0.4, 0.5, 0.8, 0.95].

***N***	**_*P*(*p* ≤ α)_**	**σ**
97	0.04	0.29
135	0.03	0.28
309	0.03	0.29
541	0.05	0.29

**Table 12:** The proportion of false substantial falsifications and false preliminary verifications using $LR\geq \frac{\left(1-\beta \right)}{\alpha }$.

**$P\left(LR\geq \frac{\left(1-\beta \right)}{\alpha }\right)$**	**Substantial falsification**	**Preliminary verification**
***N***	*$\frac{L\left(d>\delta |D\right)}{L\left(d=0|D\right)}$*	*$\frac{L\left(d=\delta |D\right)}{L\left(d=0|D\right)}$*
97	0.09	0.00
135	0.04	0.00
309	0.03	0.00
541	0.04	0.01

The preceding simulations suggest that, given the threshold $LR\geq \frac{\left(1-\beta \right)}{\alpha }$, the proportion of false negative results remains too large. One might therefore lower the threshold to $3<LR<\frac{\left(1-\beta \right)}{\alpha }$, which still indicates *some* evidence for the *H*_1_ (see Figure 2). Whether this new threshold reduces the proportion of false negative results *unproblematically* directly depends on the proportion of false positives. Compared to the case of falsification, however, we now retain a larger proportion of false positives (see Table 13 and Figure 3).

**Table 13:** The proportion of false preliminary verifications using *LR* = 3.

***N***	**$P\left(\frac{L\left(d=\delta |D\right)}{L\left(d=0|D\right)}>3\right)$**
97	0.10
135	0.09
309	0.07
541	0.00

As we combine the threshold $LR\geq \frac{\left(1-\beta \right)}{\alpha }$ with the substantial verification-criterion, the previous simulations retained a rather large proportion of false negative results. However, this increase occurs only if data are of *low* to *medium* induction quality. If induction quality approaches α = β = 0.01, by contrast, then the proportion of both false positive and false negative results decreases to an acceptable minimum. Hence, we may falsify the *H*_0_ and *simultaneously* verify the *H*_1_.

### Simulation 4

#### Purpose

Simulations 1–3 confirmed a simple relation: increasing induction quality of data decreases the proportion of false positive results. Where an actual experimental manipulation fails to produce its expected result, this relation may now tempt researchers to *post-hoc* manipulate induction quality of data, by shedding some of the “failing” data-points. Simulation 4 investigates the consequences of this move.

#### Method

Using the samples from Simulation 4, we remove from each sample the 10% of data that score lowest on the dependent variable, thus least support the *H*_1_, and then re-assess the proportion of false positive findings.

#### Results and discussion

Rather than increase induction quality of data, this *post-hoc* manipulation produces the opposite result: it raises the proportion of false positive results. On all of our criteria, indeed, shedding the 10% of data that least support the focal hypothesis increases the error-rates profoundly (see Table 14).

**Table 14:** The proportion of false substantial falsifications and false preliminary verifications, given one had obtained a preliminary discovery (as per the *p*-value and *LR*), after 10% of least hypothesis supporting data were removed.

		$P\left(LR\geq \frac{\left(1-\beta \right)}{\alpha }\right)$	
		**Substantial falsification**	**Preliminary verification**
***N***	***P*(*p* ≤ α)**	***$\frac{L\left(d>\delta |D\right)}{L\left(d=0|D\right)}$***	***$\frac{L\left(d=\delta |D\right)}{L\left(d=0|D\right)}$***
87	0.36	0.24	0.19
121	0.40	0.25	0.18
287	0.80	0.58	0.47
487	0.95	0.87	0.80

Published data, of course, do not reveal whether someone shed parts of them. Where this manipulation occurs but one cannot trace it reliably, this risks that others draw invalid inferences. For this reason alone, sound inferences should rely on the *aggregate* results of independent studies (This assumes that data shedding is not ubiquitous). As RPS's favored aggregation method, we therefore simulate a log-likelihood-addition of such results.

### Simulation 5

#### Purpose

We generally advocate *high* induction quality of data. Collecting the sizable *N*_MIN_ (that particularly laboratory studies require) to meet test-power = 0.99 (or merely 0.95), however, can quickly exhaust an individual research group's resources (see Lakens et al., 2018)^8^. In fact, we often have no other choice but to aggregate comparatively “soft” (underpowered) data from multiple studies. Aggregate data, of course, must reflect the trust each dataset deserves individually. We therefore simulate the addition of logarithmic *LR*s (log-*LR*s) for data of low to medium test-power.

#### Method

We add the log-*LR*s as per the low-powered samples of Simulation 1, then assess the proportions of samples that meet the criteria of each RPS-step. Notice that this is the only way to conduct a *global* hypothesis-test that combines individual studies safely (It is nevertheless distinct from a viable meta-analytic approach; see Birnbaum, 1954).

#### Results and discussion

Table 15 shows that the log-*LR*s of three *low-powered* studies under (1–β) = [0.4, 0.5, 0.8] aggregate to one *medium-powered* study under (1–β) = 0.95 (see Table 3), because the three samples sum to *N*_MIN_ for a substantial discovery under (1–β) = 0.95. The probability of correctly rejecting the *H*_0_ thus approaches 1, whereas the proportion of preliminary verifications is not much larger than for each individual study (see Table 3; last row). This means individual research groups can collect *fewer* data points than *N*_MIN_. Thus, log-*LR* addition indeed optimizes a substantial *H*_0_-falsification.

**Table 15:** The proportions of $LR\geq \frac{\left(1-\beta \right)}{\alpha }$ when adding the log(*LR*) of individually underpowered studies featuring (1–β) = [0.4, 0.5, 0.8].

$P\left(\sum \log(LR)\geq \log\left(\frac{\left(1-\beta \right)}{\alpha }\right)\right)$	***δ***			
	**0.01**	**0.2**	**0.5**	**0.8**
Preliminary verification $\frac{L\left(d=\delta |D\right)}{L\left(d=0|D\right)}$	0.71	0.73	0.75	0.74
Substantial falsification $\frac{L\left(d>\delta |D\right)}{L\left(d=0|D\right)}$	0.99	1	1	1

Simulations 1–5 recommend RPS primarily for its desirably low error-rates, which to achieve made induction quality of data and likelihood-ratios central. Particularly Simulation 5 shows why log-likelihood-ratio addition of individually under-powered studies can meet the rigorous test-power demands of the justification context, viz. (1–β) = 0.95, or better yet (1–β) = 0.99.

## Discussion

As an alternative to testing the *H*_1_ against *H*_0_ = 0, we may pitch it against *H*_0_ = random. Following a reviewer's suggestion, we therefore also simulated testing the mean-difference between the treatment- and the control-group against the *randomly varying* mean-difference between the control-group and zero. Compared to pitching the *H*_1_ against *H*_0_ = 0, this yields a reduced proportion of false negatives, but also generates a higher proportion of false positives.

Since our sampling procedure lets the mean-difference between control group and zero vary randomly around zero, the increase in false positives (negatives) arises from the control group's mean-difference falling *below* (*above*) zero in roughly 50% of all samples. This *must* increase the *LR* in favor of the *H*_1_ (*H*_0_). With respect to comparing group-means, however, testing the *H*_1_ against *H*_0_ = random does *not* prove superior to testing it against *H*_0_ = 0, as in RPS.

In view of RPS, if induction quality of data remains *low* (α = β > 0.05), then we cannot hope to either verify or falsify a hypothesis. This restricts us to two discovery context-activities: making a *preliminary* or a *substantial discovery* (RPS-step 1, 2). After all, since both discovery-variants arise from estimating *p*(*D*,*H*), this rules out hypothesis-testing research, which instead estimates *L*(*H*|*D*).

By contrast, achieving *medium* induction quality (α = β ≤ 0.05) meets a crucial precondition for justification context-research. RPS can now test hypotheses against “hard” data by estimating *L*(*H*|*D*). Specifically, RPS tests a *preliminary*, respectively a *substantial H*_0_*-falsification* (RPS-steps 3, 4), by testing if $\frac{L\left(d>0|D\right)}{L\left(d=0|D\right)}$, respectively $\frac{L\left(d=\delta |D\right)}{L\left(d=0|D\right)}$, exceeds $\frac{\left(1-\beta \right)}{\alpha }$. If the latter holds true, then we can test a *preliminary verification* of the theoretical effect-size *H*_1_-hypothesis (RPS-step 5) as to whether $\frac{L\left(d=\delta |D\right)}{L\left(d=0|D\right)}$ exceeds $\frac{\left(1-\beta \right)}{\alpha }$. If so, then we finally test a *substantial H*_1_*-verification* (RPS-step 6)—here using the ratio of the MLE of data and the likelihood of the *H*_1(*d*=δ)_—as to whether δ falls within the 95%-interval centered on the MLE [If not, we may adapt *H*_1(*d*=δ)_ accordingly, provided both theoretical and empirical considerations support this].

As we saw, RPS almost eliminates the probability of a false positive *H*_1_-verification. If data are of medium induction quality, moreover, then the probability of falsely rejecting the *H*_1_ lies in an acceptable range, too (This range is even slightly smaller than that for false positive verifications). However, lowering the threshold $\frac{\left(1-\beta \right)}{\alpha }$ to decrease the probability of false negatives *will* increase the probability of false positives. In balancing false positive with false negative *H*_1_-verifications, then, we face an inevitable trade-off.

To increase the probability of false positives is generally more detrimental to a study's global outcome than to decrease the probability of false negatives. After all, since editors and reviewers typically prefer significant results (*p* < α = 0.05), non-significant results more often fail the review process, or are not written-up (Franco et al., 2014). This risks that the community attends to more potentially false positive than potentially false negative results^9^. That researchers *should* reduce this risk thus speaks decisively against lowering the threshold. To control the risk, moreover, it *suffices* to increase induction quality of data by adding additional samples until *N* = *N*_MIN_.

In psychology as elsewhere today, the standard mode of empirical research clearly differs from what RPS recommends; particularly induction quality (test-power) appears underappreciated. Yet, what besides a substantial *H*_1_-verification can provide a statistical warrant to accept a *H*_1_ that aptly pre- or retrodicts a phenomenon? Likewise, only a substantial *H*_0_-falsification can warrant us in rejecting the *H*_0_ (For reasons given earlier, *p*-values alone will not do).

The discovery context as RPS's origin and the justification context as its end, RPS employs empirical knowledge to gain theoretical knowledge. A theory is generally more informative the more possible states-of-affairs it rules out. The most informative kind of theory, therefore, lets us deduce hypotheses predicting *precise* (point-specified) empirical effects—effects we can falsify statistically^10^. Obvious candidates for such point-values are those effects that “hard” data support sufficiently. RPS's use of statistical inference toward constructing improved theories thus reflects that, rather than one's statistical school determining the most appropriate inference-element, this primarily depends on the prior state of empirical knowledge we seek to develop.

Such prior knowledge we typically gain via meta-analyses that aggregate the samples and effect-sizes of topically related object-level studies. These studies either estimate a parameter or test a hypothesis against aggregated data, but typically are individually underpowered. A meta-analysis now tends to *join* an estimated combined effect-size of several studies, one on hand, with the estimated sum of their confidence intervals deviating from the *H*_0_, on the other. This aggregate estimate, however, thus rests on data of *variable* induction quality. A similar aggregation method, therefore, can facilitate only a parameter-estimation, but it will not estimate *L*(*H*|*D*) safely.

A typical meta-analysis indeed *ignores* the replication-probability of object-level studies, instead considering only the probability of data, *p*(*D*,*H*)^11^. This makes it an instance of discovery context-research. By contrast, log-likelihood-addition is *per definition* based on trustworthy data (of high induction quality), does estimate *L*(*H*|*D*) safely, and hence is an instance of justification context-research (see sections Three Measures and Discussion).

RPS furthermore aligns with the registered replication reports-initiative (RRR), which aims at more realistic empirical effect-size estimates by counteracting *p*-hacking and publication bias (Bowmeester et al., 2017). Indeed, RPS *complements* RRR. Witte and Zenker's (2017a) re-analysis of Hagger et al.'s (2016) RRR of the ego-depletion effect, for instance, strengthens the authors' own conclusions, showing that their data lend some 500 times more support to the *H*_0(*d*= 0.00)_ than to the *H*_1(*d*=0.20)_.

Both RRR and RPS obviously advocate effortful research. Though we *could* coordinate such efforts across several research groups, current efforts are broadly individualistic and tend to go into making *preliminary discoveries*. This may yield a more complex view upon a phenomenon. Explaining, predicting, and intervening, however, all require theories with substantially verified *H*_1_-hypotheses as their deductive consequences. Again, constructing a more precise version of such a theory is RPS's main aim. Indeed, we need *something like* RPS anyways. For we can statistically test hypotheses by induction [see section The Research Program Strategy (RPS)], but we cannot outsource theory-construction to induction.

## Frequentism vs. bayesianism vs. RPS

A decisive evaluative criterion is whether an inference strategy leads to a rigorously validated, informative theory. Researchers can obviously support this end only if their individual actions relate to what the research community does as a whole. At the same time, each researcher must balance her own interests with those of others. Hence, we exercise “thrift” when collecting small samples, but also publish the underpowered results this generates to further our careers.

Reflecting the research community's need for informative theories, most journals require that a submitted manuscript report at least one statistically significant effect—that is, a *preliminary discovery* à la NHST (For an exception, see Trafimow, 2014). Given this constraint, the favored strategy to warrant our publication activities seemingly entails conducting “one-shot”-experiments, leading to many papers without integrating their results theoretically.

That strategy's probably best defense offers three supporting reasons: (i) the strategy *suffices* to discover non-random effects; (ii) non-random effects *matter* in constructing informative theories; (iii) the *more* such discoveries the merrier. However, (i) is a necessary (rather than a sufficient) reason that the strategy is apt; (ii) is an insufficient supporting reason, for non-randomness matters but test-power counts (Witte and Zenker, 2018); and (iii) obviously falls with (ii). Therefore, this defense cannot sufficiently support that the strategy balances the interests of all concerned parties. Indeed, the *status quo* strongly favors the individual's career aspirations over the community's need for informative theories.

The arguably best *statistical* method to make a discovery remains a Fisher-test (For other methods, see, e.g., Woodward, 1989; Haig, 2005). It estimates the probability of an empirical effect given uncontrollable, but non-negligible influences. This probability meeting a significance-threshold such as *p*(*H*,*D*) < α = 0.05, as we saw, is a necessary and sufficient condition for a *preliminary discovery* (RPS-step 1). Though this directs our attention to an empirical object, it also exhausts what NHST by itself can deliver. Subsequent RPS-steps therefore employ additional induction quality measures, namely the effect-size (steps 2–5) and offer a new way of using confidence intervals (step 6).

Recent critiques of NHST give particular prominence to Bayesian statistics. As an alternative to a classical *t*-test, for instance, many promote a Bayesian *t*-test. This states the probability-ratio of data given a hypotheses-pair, *p*(*D*|*H*_1_)/*p*(*D*|*H*_0_), a ratio that is known as the “Bayes factor” (Rouder et al., 2009; Wetzels et al., 2011). If the prior probabilities are identical, *p*(*H*_1_) = *p*(*H*_0_) = 0.50, then the Bayes factor *is* the likelihood-ratio of two point-hypotheses, *L*(*H*_1_|*D*)/*L*(*H*_0_|*D*). Indeed, RPS largely is coextensive with a Bayesian approach as concerns the *hypothesis space*.

But Bayesians must also operate in the *data space*, particularly when selecting data-distributions as priors for an unspecified *H*_1_. Such substantial assumptions obviously demand a warrant. For the systematic connection between the Bayes-factor and the *p*-value of a classical *t*-test is that “default Bayes factors and *p*-values largely covary with each other” (Wetzels et al., 2011, 295). The main difference is their calibration: “*p*-values accord more evidence against the null [hypothesis] than do Bayes factors” (ibid).

The keyword here is “default.” For the default prior probabilities one assumes matter when testing hypotheses. In fact, not only do Bayesians tend to assign *different* default priors to the focal *H*_0_ and the *H*_1_; they also tend to *distribute* (rather than point-specify) these priors. As Rouder et al. (2009, 229) submit, for instance, “[…] we assumed that the alternative [hypothesis] was at a single point”—an assumption, however, which allegedly is “too restrictive to be practical” (ibid). Rather, it be “more realistic to consider an alternative [hypothesis] that is a distribution across a range of outcomes” (ibid), although “arbitrarily diffuse priors are not appropriate for hypothesis testing” (p. 230) either. This can easily suggest that modeling a focal parameter's prior probability *distributively* would be the innocent choice it is not.

After all, computing a Bayesian *t*-test necessarily incurs not only a specific prior data-distribution, but also a point-specified scaling factor. This factor is given by the prior distributions of the focal hypotheses, i.e., as the ratio *p*(*H*_1_)/*p*(*H*_0_) [see our formula (1), section Three Measures]. *Prior* to collecting empirical data, therefore, *p*(*H*_1_)/*p*(*H*_0_) < 1 reflects a (subjective) bias *pro* the *H*_0_–which lets data raise the ratio's denominator—while *p*(*H*_1_)/*p*(*H*_0_) > 1 reflects a preference *contra* the *H*_0_.

If the priors on the *H*_0_ and the *H*_1_ are unbiased, by contrast, then the scaling factor “drops out.” It thus qualifies as a hidden parameter. Alas, unbiased priors are the exception in Bayesian statistics. A default Bayesian *t*-test, for instance, normally assumes both a Cauchy distribution and a scaling factor of 0.707. Both assumptions are of the *same strength* as the assumptions that RPS incurs to point-specify the *H*_1_. The crucial difference, however, is that the two Bayesian assumptions concern the *data space*, whereas RPS's assumptions pertain to the *hypotheses space*.

Unlike RPS's assumptions, the two Bayesian assumptions thus substantially influence the shape of possible data. For the scaling factor's value grounds in the type of the chosen prior-distribution, which hence lets the Bayes factor vary noticeably. Different default priors can thus lead to profound differences as to whether data corroborate the *H*_0_- or the *H*_1_-hypothesis

Moreover, a Bayesian *t*-test's result continues to depend on the sample size, and lacks information on the replication-probability of data given a true hypothesis.

The most decisive reason against considering a standard Bayesian approach an all-things-considered *best* inference strategy, finally, is that it remains unclear how to sufficiently justify this or that scaling factor, or distribution, not only “*prior* to analysis[, but also] *without* influence from [sic] data” (Rouder et al., 2009, 233; *italics added*). Indeed, the need to fix a Bayesian t-test's prior-distribution *alone* already fully shifts the decision—as to the elements an inference strategy should (not) specify—from the hypotheses space to the data space. This injects into the debate a form of subjectivity that point-specifying the *H*_1_ would instead make superfluous.

One should therefore treat a Bayesian *t*-test with utmost caution. For rather than render hypothesis testing simple and transparent, a Bayesian *t*-test demands additional efforts to bring its hidden parameters and default priors *back into view*. We would hence do well to separate our data exploration-strategy clearly from our hypothesis-testing machinery. The Bayesian approach, however, either would continue *not* to mark a clear boundary or soon look similar to RPS's hybrid-approach^12^.

To summarize the advantages RPS offers over both a pure Frequentist and a standard Bayesian approach:

- RPS uses NPTT to determine the minimum sample size, *N*_MIN_, that suffices to conduct research under at least medium induction quality of data (α = β < 0.05);
- the RPS hypothesis corroboration-threshold is sensitive to both errors (α, β);
- to facilitate an aggregate hypothesis-evaluation (balancing resource restrictions with career aspirations), RPS uses log-likelihood-addition to integrate individually underpowered studies.

RPS thus makes explicit why a statistical result depends on the sample-size, *N*. Using a point-alternative hypothesis particularly shows that the Bayes-factor varies with *N*, which otherwise remains “hidden” information. Throughout RPS's six steps, the desirably transparent parameter to guide the acceptance or rejection of a hypothesis (as per Wald's criterion) is induction quality of data (test-power).

Finally, notice that the “new statistics” of Cumming (2013) only pertains to the data space. As does Benjamin et al.'s (2018) proposal to lower α drastically. For it narrowly concerns a preliminary discovery (RPS-step 1), but leaves hypothesis-testing unaddressed (also see Lakens et al., 2018). To our knowledge, no equally appropriate and comprehensive strategy currently matches the inferential capabilities that RPS offers (Wasserstein and Lazar, 2016).

## Conclusion

RPS is a hybrid-statistical approach using tools from several statistical schools. Its six hierarchical steps lead from a preliminary *H*_1_-discovery to a substantial *H*_1_-verification. Each step not only makes a prior empirical result from an earlier step more precise, our simulations also show that completing RPS's six steps nearly eliminates the probability of false positive *H*_1_-verifications. If data are of medium induction quality, moreover, then also the probability of falsely rejecting the *H*_1_ lies in an acceptable range.

Having simulated a broad range of focal parameters (α, β, *d, N*), we may extrapolate to implicit ranges safely. This lets us infer the probable error-rates of studies that were conducted independently RPS and thus allows estimating how trustworthy a given such result is. The online-tool we supply indeed makes this easy.

We advocate RPS primarily for the very low error-rates of its empirical results (Those feeling uncertain about such RPS-results may further increase the sample, to obtain yet lower error-rates). Moreover, an integration of individually underpowered studies via log-likelihood-addition not only is meaningful, it can also meet the test-power demands of the justification context. Therefore, research groups may cooperate such that each group collects fewer that the minimum number of data points.

Null-hypothesis significance testing *by itself* can at most deliver a preliminary discovery (RPS-step 1). This may motivate new research questions, which for RPS is merely an intermediate goal; the aim is to facilitate theory development and testing. Since most current research in psychology as elsewhere stops at RPS-step 1, however, this cannot suffice to construct well-supported and informative theories. Indeed, that an accumulation of preliminary discoveries could lead to a well-supported theory ever remains a deeply flawed idea.

## Author contributions

The idea for RPS originates with EW. FZ and EW jointly developed its presentation. AK-S programed and ran the simulations. EW wrote the first draft of the manuscript; all authors edited it and approved the final version.

### Conflict of interest statement

The authors declare that the research was conducted in the absence of any commercial or financial relationships that could be construed as a potential conflict of interest.

## Footnotes

^1^See https://osf.io/pwc26/ for the R-code; find the online-tool at https://antoniakrefeldschwalb.shinyapps.io/ResearchProgramStrategy/

^2^Our focus here is on the *quantitative* evaluation of hypotheses by empirical data. The current presentation of RPS therefore both excludes qualitative research processes preceding data-collection like conjecturing phenomena or constructing experimental designs (see Flick, 2014) as well as subsequent processes like embedding data into an informative theory. Both process kinds employ observation and interpretation, but also rely on scholarly argument referencing more than statistical data alone. Nonetheless, insofar as empirical data are independent of a researcher's prior belief, such data are necessary to run a research program.

^3^The smallest sample, *N*_MIN_, sufficing in NPTT to identify a point-specified effect as a statistically significant deviation from random, is a function of α, β, and *d*. Under conventional errors (e.g., α = β ≤ 0.05), therefore, given any sample, *N*, a significance-test is optimal if *N* = *N*_MIN_. With both hypothesis-verification and -falsification alike, however, if *N* > *N*_MIN_, then the utility of additional data decreases. Under α = 0.05 (one-tailed), for instance, already *N* = 500 let the very small effect *d* = 0.10 become statistically significant, even though it “explains” but 0.002% of data-variance. Once *N* > 60.000, this utility vanishes. Almost any way of partitioning a very large sample now makes virtually the smallest effect statistically significant (Bakan, 1966). This may seem paradoxical because the law of great numbers states that, *ceteris paribus*, enlarging *N* increases the validity of a parameter-estimate. At *N* > 60,000, however, measuring virtually *any* variable “reveals” that it significantly deviates from some predicted value. In a statistical sense, all unknown influences can now sufficiently manifest themselves, which lets *any* parameter-value become equally admissible. But if every parameter could become statistically significant, then none would be particularly important. *Ad absurdum*, then, as concerns hypothesis-testing the claim “more data is always better” is false *in the hypotheses space*. It nevertheless holds that increasing the sample yields an ever more precise parameter-estimate *in the data space*.

^4^Fixing the *H*_0_-parameter as *d* = 0, as a random-model has it, is merely a convention, of course. In fact RPS can alternatively base the *H*_0_-parameter on a control-group (as is typical), or on a simpler model (that eliminates elements of a more complex model), or on a rivaling theoretical model. In any case, not to remain ignorant of α, β, *N, d*, we *must* specify the *H*_0_.

^5^Witte and Zenker (2017b) presented the second and third measure as if they were one.

^6^Using “log” to abbreviate the *logarithmus naturalis* (ln), as per the command in R, in previous work we used “log” to abbreviate the *logarithmus decimalis* (Witte and Zenker, 2017b). Results are independent of nomenclature, of course, except that the critical values then came to 1.28 and 2.00.

^7^The term “random” is shorthand for a normalized mean, irrespective of whether we assume random influences, work with a control group or a simpler model (featuring fewer parameters), or with a theoretical alternative model.

^8^Governing the praxis of enlarging the sample until we reach sufficient test-power is the assumption that the focal theoretical parameter is the mean at the group level, whereas participant behavior at the individual level fluctuates randomly. If we instead focus on the potential non-random variation at the individual level, of course, then it is not the size of the sample (number of participants) that counts, but the number of repeated measurements we perform on a single participant. With a “small-N design,” indeed, the population is the single participant (see Smith and Little, 2018). Provided our indicators are valid and reliable, repeated measurements on a single participant may in fact detect idiosyncratic influences that averaging at the group level could distort. But rather than offer an alternative to large sample research, a small-N design serves a distinct purpose, and so complements large sample research.

^9^Things might look different if, next to a truth-criterion (based on error probabilities), we employ external utilities, too (Miller and Ulrich, 2016). Even where we can motivate such utilities unproblematically, we must ever compare the empirical proportions of simulated false positive results vs. false negative *substantial verifications*. Under medium induction quality (α = β = 0.05), the odds-ratio roughly is 1:5, under high induction quality (α = β = 0.01) it is 1:10. To compare with the proportion of *substantial discoveries* and *substantial falsifications*, under medium induction quality the odds-ratio decreases to about 1:2; under high induction quality nearly to 1:1. As we saw in the previous section, the asymmetry itself arises from comparing a point-parameter in case of *false negatives*, with a distributed-parameter (an interval) in case of *false positives*.

^10^If we are uncertain which point-hypothesis best specifies a theoretical parameter, then we may generalize the parameter from a point- to an interval-hypothesis. The interval's end-points thus state distinct (hypothetical) effect-sizes; the middle point qualifies as a theoretical assumption. To achieve *constant* induction quality, of course, we must confront each end-point with its appropriate sample size. To this end, log-likelihood-addition lets us increase the sample associated to the larger effect-size until we reach the appropriate sample-size for the smaller effect-size.

^11^Here, we can neither discuss meta-analysis as a method, nor adequately address the replication of empirical studies. We show elsewhere how to statistically establish hypotheses by integrated efforts, particularly addressing Bem's psi-hypothesis (Witte and Zenker, 2017b) and the ego-depletion effect (Witte and Zenker, 2017a).

^12^Schönbrodt and Wagenmakers's (2018) recent Bayes factor design analysis (BFDA), for instance, clearly recognizes the need to first plan an empirical setting, to only then evaluate the degree to which actual data falsify or verify a hypothesis statistically. This same need lets RPS characterize the setting via induction quality of data. While the planning stage is independent of the analysis stage, RPS's Wald-criterion not only provides a bridge between them, it also functions as a threshold with known consequences. Unlike BFDA and similar Bayesian approaches, however, RPS avoids setting subjective priors and relies solely on the likelihood-function.
